# Nanotwinned Structure-Dependent Photocatalytic Performances of the Multipod Frameworks of Cu_7_S_4_ Hollow Microcages

**DOI:** 10.3389/fchem.2020.00015

**Published:** 2020-01-24

**Authors:** Hongdan Zhang, Yang Xuan, Peng Cheng, Wenwen Ma, Zhen Zhao, Xiaoyang Liu

**Affiliations:** ^1^Institute of Catalysis for Energy and Environment, College of Chemistry and Chemical Engineering, Shenyang Normal University, Shenyang, China; ^2^State Key Laboratory of Inorganic Synthesis and Preparative Chemistry, College of Chemistry, Jilin University, Changchun, China

**Keywords:** nanotwinned structure, Cu_7_S_4_ microcages, hollow, sacrificial template, photocatalytic mechanism

## Abstract

The 14-pods Cu_7_S_4_ hollow microcages wholly exposed with nanotwinned building blocks were successfully prepared by an ethanol-assisted sacrificial Cu_2_O template approach. Its photocatalytic activity for the degradation of methylene blue (MB) was determined. The results suggest that the Cu_7_S_4_ microcages with nanotwinned building blocks possess higher catalytic activity than the Cu_7_S_4_ microcages without the nanotwinned structures, suggesting that the special nanotwinned components can improve the catalytic performance of the multipod framework. Further investigate reveals that the nanotwins inside the Cu_7_S_4_ microcages can facilite the transport of free charges, decrease the recombination of photoinduced electrons and holes, and elongate the lifetime of the electron–hole pairs. Our work will provide powerful evidence that the nanotwinned building blocks of the synthesized Cu_7_S_4_ microcages play a crucial role for the high catalytic activity.

## Introduction

It is well-known that the physical and chemical properties of a material could be significantly affected by its size, morphology, composition, crystallinity, and structure (Bruda et al., 2005; Shen et al., 2015; Ye et al., 2017). Hollow micro/nanostructures have attracted increasing attention due to their large surface areas, good surface permeability, low densities, low coefficients of thermal expansion, and refractive indexes and have shown their outstanding performances in various fields including gas sensors, biomedical diagnosis, catalysis, drug-delivery, and chemical reactors (Ding et al., 2010; Wang et al., 2010; Liu J. et al., 2011; Xia et al., 2012; Yu et al., 2013). Recently, the nanotwinned structures have become a research focus due to their large surface energies and excellent mechanical properties (Lu et al., 2004, 2009). In addition, some materials with nanotwinned structures exhibit excellent photocatalytic properties. For example, Liu M. et al. (2011) reported that the introduction of nanotwins to Cd_1−x_Zn_x_S semiconductors could significantly improve their photocatalytic activities. Chowdhury et al. (2011) demonstrated that the nanotwinned structures on TiO_2_ played a crucial role in improving the catalytic activity. Therefore, it is expected more and more potential applications of nanotwinned structures can be discovered by the introduction of nanotwinned building blocks into crystals. Building nanotwinned blocks on crystals are attractive, yet challenging. The shape-controlled synthesis of polyhedral hollow materials with nanotwinned building blocks remains a challenging topic for both fundamental study and practical applications. In addition, the photocatalytic mechanism and pathway are still ambiguous. The twins in photocatalysts with highly ordered structures can facilitate the transports of free charges in perfect crystals, while prevent the re-combination of holes and electrons (Liu M. et al., 2011). However, the evidence there still lack to support such statement. Therefore, exploring the related process mechanism is of significant importance.

Copper sulfide, a non-stoichiometric *p*-type semiconductor with the direct band gap 1.2–2.0 eV, has exhibited great potentials in various applications, such as solar cells, optical filters, nanoswitches, thermoelectric and photoelectric transformers, superconductors, sensors, and lithium-ion batteries with its unique optical, electric, and thermal properties (Grozdanov and Najdoski, 1995; Šetkus et al., 2001; Sakamoto et al., 2003; Lee et al., 2007; Lai et al., 2010). Most reports have been focused on the synthesis of copper sulfide nanocages via a sacrificial Cu_2_O template approach based on the Kirkendall effect. Various Cu_7_S_4_ cages with single-crystalline shells (such as cubes, octahedra, and multi-facet polyhedra; Cao et al., 2005; Jiao et al., 2006; Zhang et al., 2009) and meso-crystalline shells (Sun et al., 2011, 2012) are synthesized using single-crystalline cuprous oxide (Cu_2_O) crystals as sacrificial templates. However, the structure-controlled synthesis of different building blocks in Cu_7_S_4_ hollow nanocages with polycrystalline shells is rarely studied. In the present work, we have developed an ethanol assisted sacrificial Cu_2_O template approach to the shape-controlled synthesis of nanotwinned Cu_7_S_4_ microcages with Multipod Framework shell by an ethanol assisted sacrificial Cu_2_O template approach. The structure-dependent photocatalytic performances and mechanism of the hierarchical Cu_7_S_4_ hollow microcage assemblies with twinned nanoplate building blocks are determined and discussed in detail.

## Experimental

### Chemicals

The chemicals used in this study included Cu (Ac)_2_ 2H_2_O (99.5%, Guangdong xilong Co., China), EDTANa_2_ ·2H_2_O (99.0%, Nanjing Chemical Company, China), NaOH (96%, Beijing Co., China), Na_2_S·2H_2_O (99.5%, Guangdong xilong Co., China) n-butyl alcohol (99.5%, Nanjing Chemical Company, China), and ethanol (AR, Beijing Fine Chemical Company, China). All the reagents and solvents for synthesis were purchased from commercial sources and used as received without further purification.

### Synthesis of Cu_2_O Templates

The 14-pod Cu_2_O template was synthesized as described in the previous report (Zhang et al., 2015). Briefly, 0.11 g Cu (Ac)_2_·2H_2_O and 3.2 mL n-butyl alcohol were added into a clear aqueous solution containing 0.744 g EDTANa_2_·2H_2_O, 0.32 g NaOH and 6.8 mL deionized water. The mixture was sonicated for 30 s, transferred into a teflon-lined stainless steel autoclave, heated at 100°C for 5 h, and then cooled to the ambient temperature. The red products were collected and washed with distilled water and absolute ethanol, respectively, for three times.

### Synthesis of Cu_2_O–Cu_7_S_4_ Core–Shell Particles

The 14-pod Cu_2_O templates obtained above was dispersed in 100 mL anhydrous ethanol containing 0.01 M Na_2_S and 0.001 M NaOH, and the mixture was stirred for 30 min at 30°C in a water bath under the air atmosphere. The precipitate was collected by centrifugation and washed with deionized water and absolute ethanol for three times, respectively.

### Synthesis of Cu_7_S_4_ Hollow Particles

The Cu_2_O–Cu_7_S_4_ core–shell particles obtained above were immersed in 25% ammonia solution for 48 h to remove the Cu_2_O cores. The hollow particles were washed three times in deionized water and anhydrous ethanol, respectively, and dried at 60°C for 12 h in a vacuum oven.

### Characterization

The phases of Cu_7_S_4_ hollow particles were determined by X-ray diffraction (XRD, Cu Kα1 radiation, Rigaku D/max2550VB, Japan). Their morphologies and structures were imaged by scanning electron microscopy (SEM, JSM-6700F, JEOL, Japan) and transmission electron microscopy (TEM, JSM-3010, JEOL, Japan), respectively. The valence states were determined by X-ray photoelectron spectroscopy (XPS, ESCALAB 250, Thermo, USA). The UV–vis absorbance spectra of as-synthesized samples were collected on a Hitachi UH-4150 (Japan) spectrophotometer, using BaSO_4_ as reference. The UV-vis absorption spectra of MB solution were recorded on a Model 2501 PC spectrometer (Shimadzu, Japan). The electrochemical impedance spectra (EIS) were carried out in a traditional three electrode system. The prepared sample was used as working electrode, a platinum plate (99.9%) was used as the counter electrode, a saturated KCl Ag/AgCl electrode was used as the reference electrode, and 0.5 M Na_2_SO_4_ solution as the electrolyte. The photoluminescence (PL) spectra were studied on Hitachi F-7000 fluorescence spectrophotometer equipped with an excitation wavelength of 370 nm.

### Photochemical Measurements

To determine the photocatalytic activity of the obtained hollow particles, 2.5 mL H_2_O_2_ (30%, w/w) was added into 4 mg/L methylene blue (MB) solution (250 mL), and then no catalyst, 12.5 mg 14-pod Cu_7_S_4_ hollow particles without the nanotwinned structures and 14-pod Cu_7_S_4_ hollow particles with the nanotwinned structures were dispersed into the solution, respectively. The final mixture was stirred for 30 min in a dark box to reach the MB adsorption equilibrium. A 2 mL MB solution aliquot was sampled every 15 min and measured for ultraviolet-visible (UV-vis) absorption spectrum.

## Results and Discussion

### XRD Patterns

**Figure 1:**
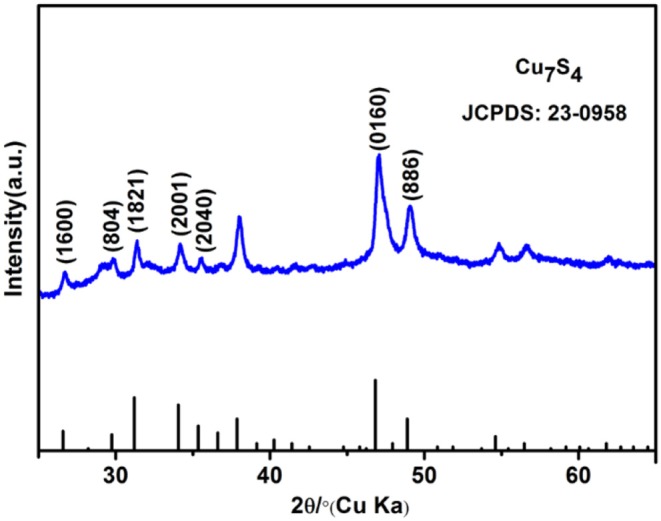
XRD patterns of the as-prepared 14-pod Cu_7_S_4_ hollow particles.

Figure 1 shows the XRD patterns of the as-prepared 14-pod Cu_7_S_4_ hollow particles. All the diffraction peaks are indexed to the standard monoclinic structure of Cu_7_S_4_. No peaks of impurities, such as copper oxides or other copper sulfides, are detected, suggesting the high purity products have been obtained.

### XPS Analysis

**Figure 2:**
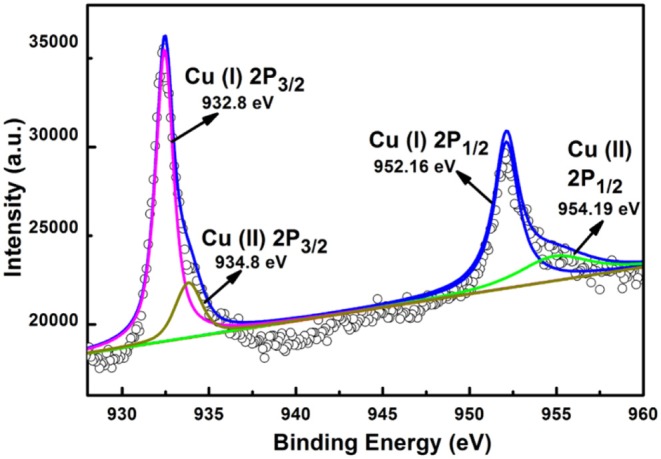
XPS spectrum of the as-prepared 14-pod Cu_7_S_4_ hollow particles.

The specific valence state of cooper in the products was determined with the electron binding energy of Cu 2p. The electron binding energies were corrected with binding energy of C1s at 284.6 eV. The 14-pod Cu_7_S_4_ hollow particles exhibit two strong XPS peaks at 932.8 and 952.16 eV, corresponding to the orbits of Cu 2p_3/2_ and Cu 2p_1/2_, respectively of Cu^+^ (Figure 2). There are two weaker peaks at 943.8 and 954.19 eV in the XPS spectrum, which correspond to the orbits of Cu 2p_3/2_ and Cu 2p_1/2_ of Cu^2+^.

### SEM Images

**Figure 3:**
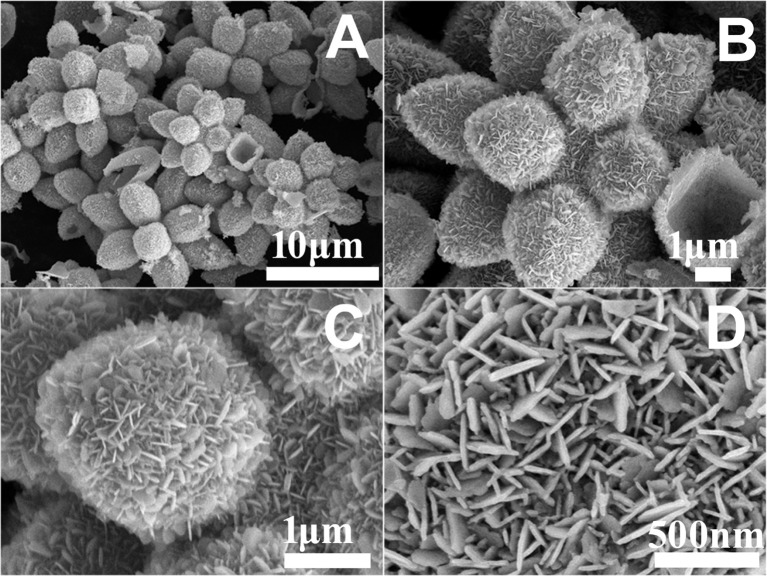
SEM images of **(A)** 14-pods Cu_7_S_4_ hollow particles. **(B)** The high magnification image of a single 14-pods Cu_7_S_4_ hollow particle. **(C,D)** A single branch and the surface of the branche of the 14-pods Cu_7_S_4_ hollow particle.

Figure 3 shows the typical SEM images of the as-prepared Cu_7_S_4_ products at different magnifications. The 14-pod Cu_7_S_4_ hollow particles are uniform and monodispersed and inherit the morphology and size of the Cu_2_O template. Its hollow structure can be found from the partially broken products. The high-magnification SEM image of a single particle reveals abundant nanoplate building blocks on the particle surface, which makes the surface very rough (Figure 3B). The enlarged SEM photographs of the single branch and the surface of the branches shown in Figures 3C,D suggest that the rough shells are composed of a large number of nanoplate building blocks, with the sizes of ~200 nm.

### TEM Images

**Figure 4:**
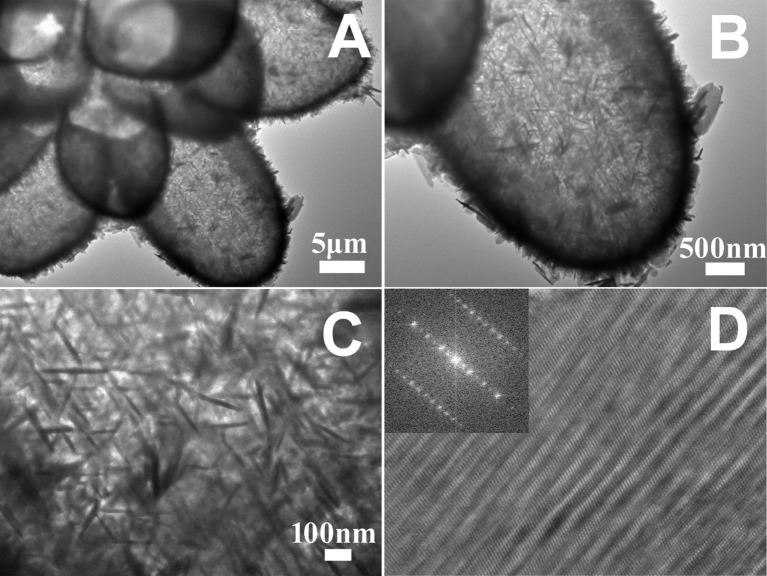
**(A)** TEM image of a 14-pod Cu_7_S_4_ hollow particle. **(B)** TEM image of a branch of the 14-pods Cu_7_S_4_ hollow particle. **(C)** TEM image of the surface of the branche of the 14-pods Cu_7_S_4_ hollow particle. **(D)** HRTEM image of single nanotwinned structure (inset: the corresponding FFT image).

The morphology and interior structure of the hollow 14-pods Cu_7_S_4_ with nanoplate building blocks were further investigated by TEM and HRTEM. Figure 4A shows a typical low-magnification TEM image of a 14-pod Cu_7_S_4_ hollow particle consisting of nanoplate building blocks. It is clear that the hollow particle has similar morphology and size as the Cu_2_O template. The TEM image of the single branches shows an extremely strong contrast between their edges (dark) and interiors (bright), which confirms the formation of hollow structures (Figure 4B). The enlarged TEM image of a single 14-pod Cu_7_S_4_ hollow particle further reveals the rich nanoplate building blocks on its surface (Figure 4C). Some regularly arranged nanoplate building blocks form the shell of the particle. Other blocks are disorderly grown on the shell of the microcages, resulting in some mesopores. Figure 4D shows the HRTEM image of the single nanoplate building blocks of Cu_7_S_4_ hollow particle. Dense defects are found on the particle surface. These undulating nanoplate building blocks may be formed from the further growth of nanoplate structures produced earlier, due to the stacking faults between lattices. The fast Fourier transform (FFT) of the HRTEM image reveals serious image tailing of the reflection fringes (Figure 4D), indicating that the nanotwinned building blocks causes the disorderedly arranged nanoplate surface.

### UV-vis Diffuse Reflectance Spectrum

**Figure 5:**
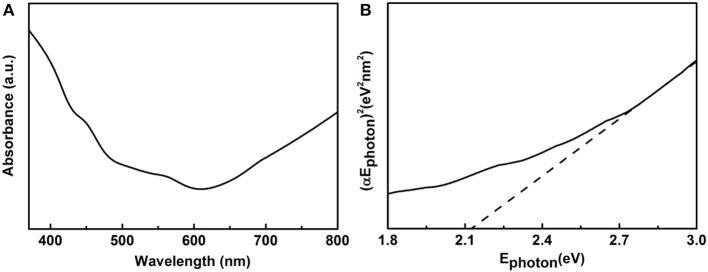
**(A)** UV-vis spectrum, and **(B)** (αEphoton)^2^ vs. Ephoton plots of Cu_7_S_4_ particles with nanotwinned structures.

UV–vis DRS was measured to further study optical absorption properties of the as prepared samples as shown in Figure 5. Figure 5A shows the UV–vis absorption spectrum of the 14-pods Cu_7_S_4_ hollow particles with nanotwinned structure. Besides a classical Tauc method was used to calculate the optical energy band gaps of above Cu_7_S_4_ materials (Tsunekawa et al., 2000). As shown in Figure 5B, the estimate value of Ephoton at α = 0 shows an absorption edge energy corresponding to Egap = 2.15 eV, which indicates the 14-pods Cu_7_S_4_ hollow particles with nanotwinned structure present visible light response.

### Photocatalytic Activity

**Figure 6:**
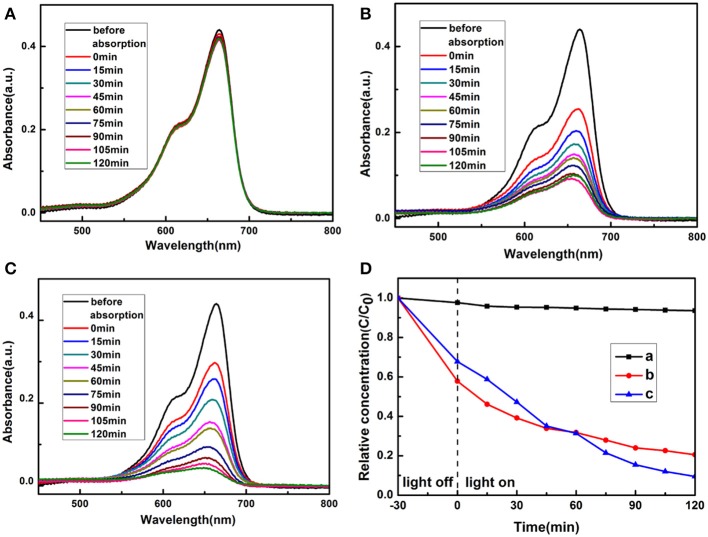
Adsorption spectra of MB solution with different catalysts **(A)** Without catalysts. **(B)** The 14-pods Cu_7_S_4_ hollow particles without nanotwinned building blocks. **(C)** The 14-pods Cu_7_S_4_ hollow particles with nanotwinned building blocks. **(D)** Extent of MB photodegradation as the function of irradiation time by different catalysts. Curve a: without catalysts; Curve b: the 14-pods Cu_7_S_4_ hollow particles without nanotwinned building blocks; Curve c: the 14-pods Cu_7_S_4_ hollow particles with nanotwinned building blocks.

To demonstrate the application potentials of the as-synthesized polyhedral 14-pods Cu_7_S_4_ hollow particles with the nanotwinned structure in the degradation of organic contaminants, their photocatalytic performance for the degradation of MB dye in the presence of hydrogen peroxide under natural light irradiation were investigated. The characteristic UV–Vis absorption of MB solution at 664 nm was monitored during the degradation to evaluate the adsorption and photocatalytic activity of the Cu_7_S_4_ hollow particles. The MB adsorption equilibrium in the dark was measured shown in Figure S1, indicating the MB adsorption equilibrium was achieved after 15 min. Figure 6 shows the peak intensities of MB solutions in the presence of different catalysts as the function of time. The adsorption of the MB solution in the absence of catalyst remains almost constant, suggesting the MB concentration does not change significantly. Therefore, the photodegradability of MB alone in solution is very poor (Figure 6A). In contrast, the UV-vis adsorption of MB solution at 664 nm decreases obviously with the irradiation time in the presence of Cu_7_S_4_ hollow particles without nanotwinned building blocks (Figure 6B), suggesting that 14-pods Cu_7_S_4_ hollow microcages can catalyze the photo-degradation of MB. However, the adsorption becomes stable in 90 min, and a large amount of MB still remains in the solution. Figure 6C shows the changes of the UV-vis adsorption of the MB solution at 664 nm with the irradiation time in the presence of 14-pods Cu_7_S_4_ hollow particles with nanotwinned building blocks. The MB is almost completely degraded, suggesting the excellent catalytic activity of the hollow particles. Figure 6D shows the plots of the absorbance of MB solutions at 664 nm vs. time, in the absence of catalyst and in the presence of 14-pods Cu_7_S_4_ hollow microcages and 14-pods Cu_7_S_4_ hollow particles with nanotwinned building blocks catalysts. The slope of the curve reflects the degradation rate. It is clear the both catalysts result in higher MB degradation rates. In addition, the MB degradation rate in the presence of 14-pods Cu_7_S_4_ hollow particles with nanotwinned building blocks is the highest.

**Figure 7:**
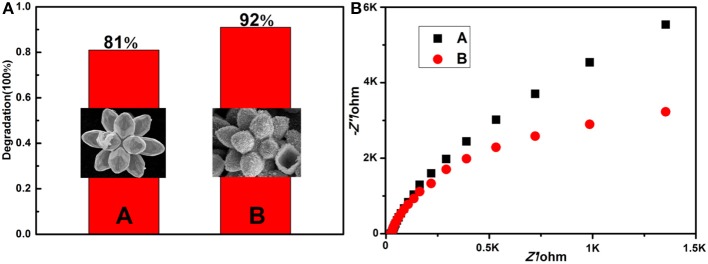
**(A)** Extent of MB photodegradation by different catalysts (inset: the corresponding SEM images). **(A)** The 14-pods Cu_7_S_4_ hollow particles without nanotwinned building blocks; **(B)** the 14-pods Cu_7_S_4_ hollow particles with nanotwinned building blocks. **(B)** Electrochemical impedance spectra (EIS).

The degradation ratios of MB solution in the first 2 h are plotted as histograms (Figure 7A), and the corresponding SEM images (the inset of Figure 7A) of Cu_7_S_4_ hollow microcages are provided. The residual ratios of MB catalyzed by 14-pods Cu_7_S_4_ hollow particles and 14-pods Cu_7_S_4_ hollow particles with nanotwinned building blocks are 19 and 8%, respectively, indicating that the later has better photocatalytic activity. The higher photocatalytic activity is possibly due to the nanotwinned structure, while further supported by electrochemical impedance analysis. As shown in Figure 7B, the 14-pods Cu_7_S_4_ hollow particles with nanotwinned building blocks show a smaller capacitive arc radius than the 14-pods Cu_7_S_4_ hollow particles without nanotwinned building blocks, which indicates the 14-pods Cu_7_S_4_ hollow particles with nanotwinned building blocks exhibit much smaller charge transfer resistance. It implies that the nanotwinned structures can improve the transfer efficient of free electrons and elongate the lifetime of electrons and holes in the photocatalytic degradation experiments, which could promote the photocatalytic activity.

### Photoluminescence Spectra

**Figure 8:**
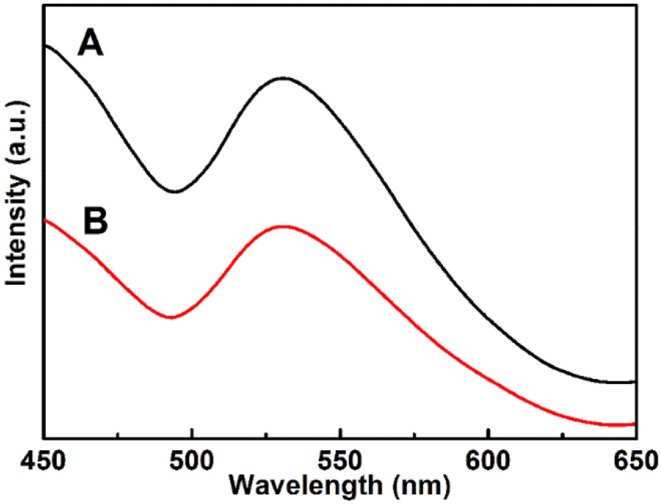
Photoluminescence (PL) spectra of different samples. **(A)** The 14-pods Cu_7_S_4_ hollow particles without nanotwinned building blocks. **(B)** The 14-pods Cu_7_S_4_ hollow particles with nanotwinned building blocks.

The photoluminescence tests of the Cu_7_S_4_ hollow particles were performed to explore the recombination of photoinduced electrons and holes, as shown in Figure 8. It can be observed that the PL spectra of both 14-pods Cu_7_S_4_ hollow particles with and without nanotwinned building blocks exhibit an emission peak at 531 nm, with excitation wavelength of 370 nm. Notably, the PL intensity of the Cu_7_S_4_ sample with nanotwinned structure decreases significantly compared with that of sample without nanotwinned building blocks, suggesting a slower recombination rate of photoinduced electrons and holes in sample with nanotwinned building blocks. The similar results are also reported in multiple literatures, for example, Shen et al. (2015); Qi et al. (2019) etc. The photodegradation and electrochemical impedance spectra of samples also confirm the above results (Figures 7A,B).

### Photocatalytic Mechanism

Based on these results, the possible photocatalytic mechanisms of the 14-pods Cu_7_S_4_ hollow particles with nanotwinned building blocks can be summarized as follows. The eletrons and holes are separated under visible light irradiation. The holes and the strong oxidant generated from the electrons are then captured by H_2_O_2_ molecules. Previously, it has been reported that the organic pollutants could be oxidized by photoinduced oxidants due to their high oxidation activity (Shi et al., 2011). The Cu_7_S_4_ hollow microcages are photolyzed by visible light, and H_2_O_2_ can enhance their photolysis. The holes in the conduction band is activated to the valence band (Wang et al., 2011), and electrons are generated in the valence band. The H_2_O_2_ molecules capture the electrons and holes to produce the strong oxidants by the following reactions (1)–(4) (Sun et al., 2012):

(1) $$\begin{array}{r} \text{C}{\text{u}}_{7}{\text{S}}_{4}+hv\to {\text{h}}_{\text{vb}}+{\text{e}}_{\text{cb}-} \end{array}$$

(2) $$\begin{array}{r} {\text{H}}_{2}{\text{O}}_{2}+{\text{h}}_{\text{vb}+}\to^{\cdot }\text{OOH}+{\text{H}}^{+} \end{array}$$

(3) $$\begin{array}{r} {\text{H}}_{\text{2}}{\text{O}}_{\text{2}}+{\text{e}}_{\text{cb}-}\to^{\cdot }\text{OH}+\text{OH} \end{array}$$

(4) $${}^{\cdot }\text{OOH}\to^{\cdot }{\text{O}}_{2}+{\text{ H}}^{+}$$

Huang et al. (2017), Chen et al. (2018), Chen et al. (2019) reported that effectively facilitating the charge separation could result in boosting oxygen activation ability substantially for producing reactive oxygen species (ROS) evolution in the photocatalytic process, which could improve photocatalytic activities. Our work has demonstrated that the nanotwinned structures of 14-pod Cu_7_S_4_ hollow particles can facilitate the transport of free electrons and improve the lifetime of electrons and holes. The 14-pods Cu_7_S_4_ hollow particles with nanotwinned building blocks exhibit a smaller capacitive arc radius and weaker peak intensity of PL than 14-pods Cu_7_S_4_ hollow particles without nanotwinned building blocks. Therefore, the nanotwinned structures greatly contribute to the high photocatalytic activity.

## Conclusions

In summary, we have successfully synthesized the multipod frameworks of Cu_7_S_4_ hollow photocatalyst with nanotwinned structures that possess superior photocatalytic activity for the degradation of MB dye. The exceptional photocatalytic activities are comprehensively attributed to the nanotwins inside the Cu_7_S_4_ hollowcages that can promote the transport of free charges, inhibit the recombination of photoinduced electrons and holes, and extend the lifetime of the electron–hole pairs. The 14-pods Cu_7_S_4_ hollow particles with nanotwinned building blocks exhibits a smaller capacitive arc radius and weaker peak intensity of PL, indicative of the promoted charge transfer, which further evidences the above conclusion. Our work is of great importance in the synthesis of high-active hollow photocatalyst with nanotwinned structures. These unique 14-pods Cu_7_S_4_ hollow cages can not only enrich the family of copper sulfide architectures, but also offers a good opportunity to understand the fundamental significance of nanotwinned structures. It is expected that hollowcages with nanotwinned building blocks can exhibit outstanding properties in various fields.

## Data Availability Statement

All datasets generated for this study are included in the article/Supplementary Material.

## Author Contributions

XL planned and supervised the project. HZ, YX, and PC performed the experiments. HZ and PC wrote the first draft. ZZ and WM revised the manuscript. XL deeply revised the manuscript.

### Conflict of Interest

The authors declare that the research was conducted in the absence of any commercial or financial relationships that could be construed as a potential conflict of interest.
