# Supplementary material for: Nanotwinned Structure-Dependent Photocatalytic Performances of the Multipod Frameworks of Cu7S4 Hollow Microcages
Source: Front Chem. 2020 Jan 24;8:15. doi: 10.3389/fchem.2020.00015 (PMC6992655; doi:10.3389/fchem.2020.00015)
Supplement: Supplementary file 1 [file Data_Sheet_1.PDF]

# Nanotwinned Structure-dependent Photocatalytic Performances of the Multipod Frameworks of $Cu_7S_4$ Hollow Microcages

**Hongdan Zhang[1], Yang Xuan[1], Peng Cheng[1*], Wenwen Ma[1], Zhen Zhao[1*] and Xiaoyang Liu[2*]**

[1] Institute of Catalysis for Energy and Environment, College of Chemistry and Chemical Engineering, Shenyang Normal University, Shenyang, China

[2] State Key Laboratory of Inorganic Synthesis and Preparative Chemistry, College of Chemistry, Jilin University, 2699 Qianjin Street, Changchun 130012, China.

***Correspondence:**
Xiaoyang Liu, liuxy@jlu.edu.cn;

Zhen Zhao, zhenzhao@cup.edu.cn;

Peng Cheng, chengp1987@126.com

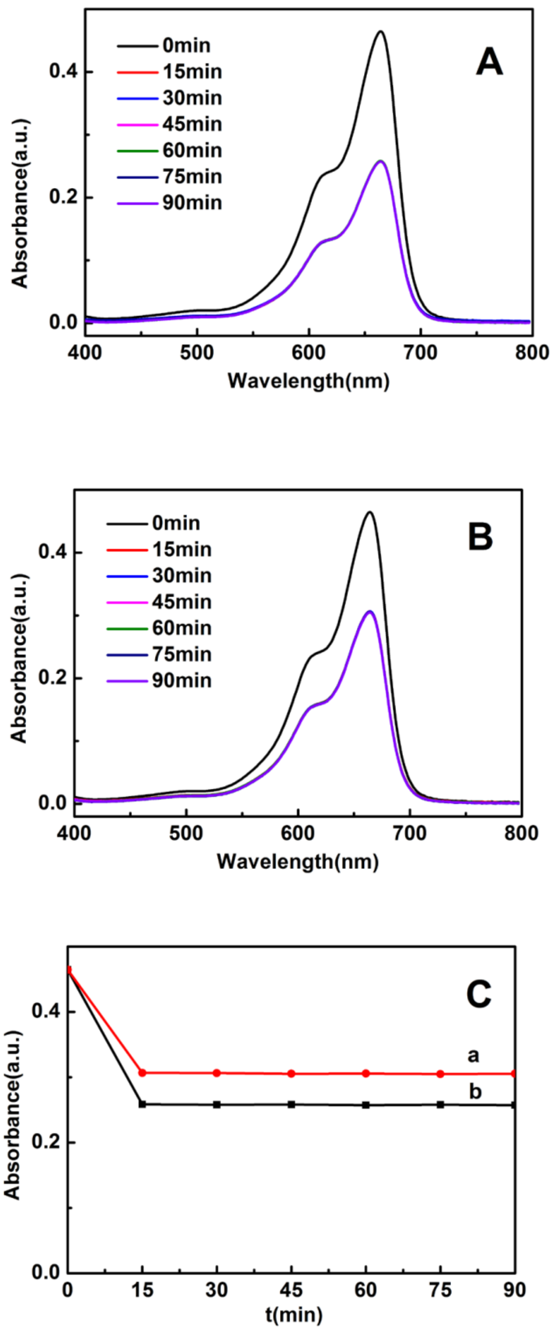


**Figure S1** UV-vis adsorption spectra of MB solution with different catalysts in a dark box. (A) The 14-pods $Cu_7S_4$ hollow particles without nanotwinned building blocks. (B) The 14-pods $Cu_7S_4$ hollow particles with nanotwinned building blocks. (C) Extent of MB adsorption in a dark box by different catalysts. Curve a: the 14-pods $Cu_7S_4$ hollow particles with nanotwinned building blocks; Curve b: the 14-pods $Cu_7S_4$ hollow particles without nanotwinned building blocks.
